# Building Strong Primary Health Care to Tackle the Growing Burden of Non-Communicable Diseases in Nepal

**DOI:** 10.1080/16549716.2020.1788262

**Published:** 2020-07-22

**Authors:** Bishal Gyawali, Pratik Khanal, Shiva Raj Mishra, Edwin van Teijlingen, Dan Wolf Meyrowitsch

**Affiliations:** a Global Health Section, Department of Public Health, University of Copenhagen, Copenhagen, Denmark; b Central Department of Public Health, Institute of Medicine, Kathmandu, Nepal; c Nepal Development Society, Bharatpur, Nepal; d Faculty of Health and Social Sciences, Bournemouth University, Poole, UK

**Keywords:** NCDs, primary healthcare, fragile health systems, integrated approach, Nepal

## Abstract

Nepal is currently facing a double burden of non-communicable diseases (NCDs) and communicable diseases, with rising trends of NCDs. This situation will add great pressure to already fragile health systems and pose a major challenge to the country’s development unless urgent action is taken. While the primary health care approach offers a common platform to effectively address NCDs through preventive and curative interventions, yet its potential is not fully tapped in Nepal. In line with the Alma Ata and Astana Declarations, we propose an integrated approach for Nepal, and other low-and middle-income countries, including six key reforms to enhance the primary care response to the increasing burdens of NCDs.

## Background

Non-communicable Diseases (NCDs) have become the leading cause of morbidity and mortality globally. Cardiovascular diseases (CVDs) account for most NCD-related deaths worldwide (17.9 million annually), followed by cancers (9.0 million), respiratory diseases (3.9 million), and diabetes (1.6 million) [1]. More than three quarters of all-cause mortality globally is attributed to NCDs, resulting in 15 million people dying between the ages of 30–69 years each year. More than three quarters of these deaths and over 85% of these premature deaths occur in low-and middle-income countries (LMICs) [1]. Like many other LMICs, in Nepal NCDs also are emerging as a major cause of morbidity and mortality [2,3]. NCDs contributed to the largest proportion of total Disability Adjusted Life Years (DALYs) and for just over half (51%) of all deaths in the country in 2018 [4]. Data from various sources reported that nearly 12% of Nepal’s adult population has Chronic Obstructive Pulmonary Diseases (COPDs) [5], 5% has cancer [6], 30% has hypertension [7], 8.4% has type 2 diabetes [8], 6% has Chronic Kidney Diseases [5], 21% are overweight/obese [9], and 23% have raised cholesterol levels [9]. The modifiable risk factors attributing to the DALYs and NCDs deaths are generally underlying behavioral, environmental and socio-economic determinants, such as inadequate physical activity, tobacco and alcohol use and unhealthy diet [9]. This increasing trend of NCDs and their related risk factors has put enormous pressure on the already fragile and challenged health systems [10], and this underlines the urgency of an innovative, comprehensive and potentially cost-effective approach to the prevention and management of NCDs.

## Primary healthcare system in Nepal

Nepal’s health system faced a drastic transformation in 2017 after the country’s administration changed from a unitary to a federal state [11]. Presently, the country has three tiers of government; one tier at the central level, provincial and the local level, respectively, with sub-national governments having more authority and resources in planning and managing health services than before [12]. The primary healthcare (PHC) system currently comprises more than 4,000 peripheral primary health facilities, including health posts, primary health centers and primary hospitals, which are managed by local level governments. Additionally, local level governments play a role in providing basic health services and conducting health promotion programmes along with prevention, control and treatment of diseases [13]. These primary health facilities under local governments provide direct services to local communities, as well as oversee community-based service delivery, mostly relying on mid-level health workers.

The Ministry of Health and Population (MoHP) at the federal level oversees functions related to regulation, quality assurance, research, outbreak control and managing specialized hospitals. Likewise, the Ministry of Social Development at the province level oversees: (1) provincial health offices; (2) hospitals providing general and specialized care; and (3) health functions of local governments, including managing logistics and drug supply [13]. In 1991, the National Health Policy laid a new foundation for PHC by establishing primary health centers in each electoral constituency and health posts in each village in order to provide access to health services for the rural and marginalized populations [14]. Similarly, the 2019 New National Health Policy aimed to establish primary hospital in each of the 753 local governments and health units in each of the 6,684 wards suggesting a progressive change in the coverage of health services [15]. Doctors, staff nurses and laboratory staffs are available at the primary health centers. At village level, services are delivered through health posts staffed by health assistants (HA), auxiliary health workers (AHWs) and auxiliary nurse midwives (ANMs) who are paid by the Government of Nepal. At the ward level, services are provided in the community by female community health volunteers (FCHVs) who are unpaid and deliver a basic package of essential maternal and child health services, including family planning, antenatal care, immunization and nutrition programmes. Additionally, AHWs, ANMs and FCHVs deliver outreach services for immunization and PHC in the community [13].

To address the emerging risk of NCDs, the MoHP launched the Package of Essential Non-Communicable Diseases (PEN) developed by the World Health Organization (WHO) in 2016 for early detection and management of CVDs, diabetes, COPD and cancer to prevent life threatening complications, such as heart attacks, stroke and kidney failure [16]. The main aim of the PEN programme was to increase access to NCD-related services in the primary health centers and health posts, the role of NCD care at the PHC level is still minimal (See Table 1).

**Table 2.** Primary health care based policy responses to NCDs.

Challenges	Primary Health Care Approach	Possible Policy Response
Children, adolescents, women of reproductive age, young and older adults are increasingly affected by risk factors for NCDs	Focus on prevention, early intervention and a life-course approach	Policies that incorporates health and social needs at all stages of the life course, including childhood, adulthood and later life and integrate into existing programmes
Critical shortages of health-care workforce, especially in rural and remote regions	CHWs as a vital component of PHC	Task shifting to CHWs, such as training FCHVs in NCD management
Caring for people with chronic care issues and multi-morbidity is challenging	Focus on engaging informal caregivers as care partners	Policies that engage, educate or support informal caregivers
Considerable inadequacies in the delivery of PHC services	Focus on sustaining, developing and strengthening PHC services	Integrating screening for NCDs into routine care using simplified guidelines; Strengthening PEN program
Weak health information systems	Focus on strengthening the evidence base for the prevention and control of NCDs	Strengthening surveillance and establishing robust integrated information management system
Financial barriers to access healthcare	A focus on UHC	Strengthening social health protection mechanism, such as health insurance and ensuring NCD services available at PHC system

**Table 1.** NCD services at various levels of PHC in Nepal [4].

S.N.	Level of health institutions	District with PEN^α^ implementation	District without PEN^α^ implementation
1	Health Post	Health education and counseling on NCD risk reduction behavior Anthropometric measurements Screening for hypertension, diabetes, breast cancer (examination) and cervical cancer as well as breast examination Drugs for hypertension, diabetes and COPD, and referral of suspected cases of NCDs	Counseling on NCD risk behavior Anthropometric measurements, Screening for hypertension and diabetes (those with laboratory), and Referral of suspected cases of NCDs
2	Primary health center	All above plus:	All above plus:
		Screening for lipid disorders (some) Early diagnosis of CVDs with the use of CVD risk prediction chart and ECG and CKDs by assessing urine protein Screening for asthma and COPD including history taking, measuring peak expiratory flow rate and managing exacerbation Management of acute presentation of CVDs Chest rehabilitation techniques	Screening for diabetes, lipid disorders (some), cervical cancer as well as breast examination Early diagnosis of CVDs and CKDs through ECG and urine protein Management of acute presentation of CVDs Drugs for hypertension, diabetes and COPD (only in health insurance implemented health facilities)
3	Primary Hospital	All above plus:	
		Screening for lipid disorders and tests for albumin, sodium and potassium Screening for breast cancer (ultrasonography) Medical management of CVD cases (outpatient, inpatient and referral)	
4	Province Hospital (former 50 bedded, zonal and regional hospital)	All above plus:	
		Medical management of CVD, hyperglycemic crises and acute renal failure cases (outpatient, inpatient and intensive care) Dialysis service (some) Biopsy test for cancer (some)	
5	Federal (Central, university and specialized hospitals)	All above plus:	
		Medical management of cases (outpatient, inpatient, intensive and interventional therapy) and specialized tests and services Renal transplant (two public and three private hospitals)	

^α^PEN programme is implemented in 50 out of 77 districts as of 2019.

## Challenges in the current health system

Similar to other LMICs, Nepal is undergoing a rapid demographic and epidemiological transition, and hence is burdened by communicable and NCDs alike [2]. Life expectancy has increased from 62.5 years in 2000 to 70.2 years in 2016 [17] and also the diseases related to aging and life conditions [18]. Healthcare is far from equitable and affordable; government spending on healthcare is very low and for NCDs specifically, and is unevenly distributed across the country. Healthcare is mainly financed by out-of-pocket expenditure, which is particularly difficult to bear for the poor people and this contributes to pronounced inequities in health. Some of the other challenges include a weak national health information and research system, interrupted supply of essential drugs for NCDs, lack of facilities and capacity for screening, early diagnosis and effective management of NCDs within the PHC system, and shortage and retention of health workforce, especially in rural areas and lack of retention. These issues have resulted in significant challenges to effectively tackle the growing burden of NCDs in Nepal. The establishment of an NCD and Mental Health Section under the Department of Health Services is a positive development. However, efforts are needed for integration of NCD services in the mainstream care with strong focus on expanding community-level services for addressing NCDs.

## Solutions to overcome challenges in primary healthcare system

Evidence has increasingly highlighted the role of the PHC approach in effectively and equitably addressing NCDs [19]. The WHO has identified PHC as an essential part of health with the implementation of Alma Ata Declaration of Alma Ata in 1978 [20] and the Astana Declaration in 2018 [21]. The Alma Ata Declaration defined PHC as ‘essential health care based on practical, scientifically sound and socially acceptable methods and technology made universally accessible to individuals and families in the community through their full participation and at a cost that the community and country can afford to maintain at every stage of their development in the spirit of self-reliance and self-determination’ [20]. Four decades later, the Astana Declaration affirmed the commitment to the fundamental right of every human being to the enjoyment of the highest attainable standard of health without distinction of any kind, and reaffirmed the commitment to the Alma Ata core principles [21]. The Declaration has recognized the increasing importance of NCDs and their common risk factors, including tobacco use, unhealthy diets, insufficient physical activity and the harmful use of alcohol and the role of PHC in the prevention, control and management of these conditions. Furthermore, the Declaration has incorporated ‘Universal Health Coverage (UHC)’ that is at the center of the health-related United Nation’s (UN) Sustainable Development Goal (SDG) 3. The PHC approach can provide a wider opportunity for health promotion, disease prevention, treatment and rehabilitation for the most prevalent NCDs, especially in resource constraint settings through mobilization of trained mid-level health workers, and all types of volunteers, families, friends, neighbors and caregivers, yet its potential has not been fully applied in Nepal. The new National Health Policy of Nepal 2019 [15] and the Multi-Sectoral Action Plan for NCD Prevention and Control (2014–2020) [22] have highlighted some aspects of prevention, control and NCD management. A previous study identified that Nepal’s NCD policies provide a ‘patchwork’ reference to international NCD policies, however, lack a clear mechanism to plan, implement and evaluate these policies locally [23]. Moreover, some NCDs-related policies have been prioritized (e.g. behavioral change communication) over others (e.g. food reformulation, taxation), possibly due to heavy resistance from corporate community. NCD policies are essentially the core part of primordial/primary prevention – lacking asymmetry in implementation of primordial/primary prevention compared to tertiary prevention has put even more pressure on the country’s already-weak PHC systems. Nepal is falling severely short of making PHC accessible and effective, particularly in the rural and remote areas where people still lack access to essential health services. For Nepal to better address NCDs, its healthcare system will need to be redesigned with a strong emphasis on PHC approach. In line with both WHO Declarations, we, propose six key reforms to promote an integrated PHC approach to NCDs in Nepal.

(1) **Life-course approach to addressing NCDs**

Social and commercial determinants of health are fundamentally interwoven into early life stages and can be linked to one’s risk of chronic disease across a life course. Along Nepal’s demographic and epidemiological transition, a third transition on diet i.e. nutritional transition is happening. A high calorie and energy dense foods, sugar sweetened beverages and alcohol are often seen as symbols of urban culture, which has penetrated the most remote corners of the country. Multiple cross-sectional studies over the past decades highlighted shifts in dietary preferences along with popularization of alcohol through digital advertising and corporate sponsorships [24]. The 2019 report on assessment of NCDs, considering large population-based samples obtained from 72 districts of the country, found that 35% of the respondents were ever drinkers, and among them nearly 71% were current drinkers, which suggests a massive shift in use of alcohol in recent years [5]. Furthermore, a recent study conducted among children aged 1–2 years in Kathmandu, Nepal’s largest city, revealed the consumption of unhealthy snacks and beverages were high among young children leading to inadequate micronutrient consumption [25]. Given Nepal’s already high rates of malnutrition, low calorie diet and sugar sweetened beverages could adversely affect child’s growth outcomes. Although, the National Health Policy 2019 and the Action Plan for NCD Prevention and Control (2014–2020) have highlighted aspects of prevention, control and NCD management, these do not fully address the importance of all age groups, type 1 diabetes and a gender-specific approach to NCDs. The latter, for example, should include the management of gestational diabetes mellitus and pregnancy-induced hypertension to improve the health of women in later life. This limits the opportunities to improve the health of the maximum number of women and their children in the most effective ways possible. A model to address NCDs at PHC level would need to include a comprehensive life-course approach to ensure that management of NCDs starts at the earliest possible stage of life [26]. The Declaration of Astana also invokes the critical need to transform some of the major successes from vertical disease-focused programmes to the delivery of comprehensive integrated care through high-quality PHC services across the life course addressing major global health threats, including NCDs through comprehensive preventive, promotive curative, rehabilitative and palliative care [21]. In this regard, interventions targeting risk behaviors, such as unhealthy diet, physical inactivity and tobacco and alcohol use should be integrated into the already existing programmes and activities at community level, such as outreach services, FCHVs and mothers’ group meetings and schools. In addition to providing routine immunizations at community, outreach services can promote early detection of NCDs through screening, effective referral and increasing awareness of NCD risk factors. Schools can be common settings to promote and reinforce healthy behaviors to prevent and reduce NCD risk factors among children and adolescents. A life-course health education can be launched in schools as part of the curriculum. FCHVs and mothers’ group can be useful to provide a preconception education to women of reproductive age, highlighting physical health, nutrition and life conditions as risk factors for NCDs. Adopting such an approach not only has implications for the way a person’s health is considered; it helps to develop appropriate and effective strategies for prevention and control of NCDs over the life course and preventing their transmission from generation to generation.

(2) **Task shifting for NCD risk factor management**

Most NCDs in Nepal are managed in tertiary health facilities which add an extra workload on the already overburdened staff. In the light of the increasing burden of NCDs and shortage of health workforce in the country, there is a need to introduce an affordable and effective strategy of managing NCDs in a PHC setting by task shifting. This was most notably reflected in the Alma Ata Declaration which envisioned community health workers (CHWs) as representing a pragmatic way of enhancing community involvement in healthcare delivery [21]. Indeed, involving CHWs have become progressively recognized as an effective and efficient primary prevention strategy for NCD management, particularly in resource-limited settings [27]. The Government of Nepal introduced a cadre of FCHVs in 1988, which has been identified as a successful strategy to address health problems at community level [28]. The FCHVs are local women often with limited formal education serving voluntarily with the government system of Nepal and considered as the frontline community health workers. They are the first point of contact between community members and PHC facilities. Around 50,000 FCHVs contribute to key public health programmes in Nepal, and are often credited for their ability to deliver and bring desired outcomes in maternal, child and preventive health in rural Nepal. Though the work modality, and work load of FCHVs is debatable, recent evidence has demonstrated that FCHVs may be effective in helping tackle the burden of hypertension in Nepal [29]. Another study also reported that devolving some NCD-related activities, including (i) health promotion for reduction of risk factors for NCDs; (ii) screening of blood pressure and blood glucose using simple non-invasive tools; (iii) referral for those who are at high risk of NCDs to the nearest health facility; (iv) education on importance of regular use of medication; and (v) recording, reporting and follow-up is potentially feasible [30]. The FCHV-based intervention supported by the PHC system could play an important role in NCD management in rural Nepal. The prospects of involving FCHVs in NCD management is encouraging, but are equally coupled with challenges, such as lack of funding for the sustainability of FCHV programmes, low literacy level, overburdened with work and lack of remuneration. A substantial investment in workforce management, logistics, training and supervision is, however, required. Further, expansion of community-based intervention should go hand-in-hand with strengthening of PHC system. Linking FCHVs with facility-based health workers and ensuring a right skill-mix between these health workers with differing level of expertise necessitates further research.

(3) **Strengthening informal care givers**

The health system in Nepal face challenges to provide care and support for people with NCDs [31]. Especially as many people with chronic care issues and multi-morbidity are also likely to have long-term care needs. The problem is particularly severe in rural areas of Nepal, where more than 80% of the population lives [31]. Informal care, which provides better coordination and closer alliances for people with chronic conditions, is a significant yet undervalued resource. Family members, friends, neighbors and people with a similar disease could potentially provide effective support for people living with chronic conditions by supporting day-to-day self-management through emotional support, direct assistance with tasks, such as taking medications and maintaining healthy diet, tracking indicators of their health status, and facilitation of healthy behaviors as they are often first and most important point of call by people in search of health services [32,33]. It is evident that more than half of adults with diabetes or heart diseases regularly involve family members in day-to-day disease management tasks [34]. However, these informal support groups have been an overlooked as source of support and there is lack of programmes to engage these informal support groups in chronic disease management [31]. Engaging FCHVs and training support groups to deliver self-management support, in collaboration with PHC teams is a promising approach to improve the quality of life of individuals with chronic conditions. These groups after receiving training may provide education, emotional support and practical problem-solving assistance. This approach has been shown to be successful with people from various communities and for specific groups of patients, such as those with cancer, diabetes and depression [32]. Furthermore, this is also grounded in the values and principles of PHC as outlined in the WHO’s Declaration of Alma Ata that the needs of patients and their families must be the main drivers of healthcare delivery [20]. However, due to an ongoing need for training and coordination as well as logistical issues, these groups may face challenges to their sustainability.

(4) **Strengthening quality of PHC and health systems**

Nepal is struggling to organize quality care for a large NCD-affected population as there remain considerable inadequacies in the delivery of PHC services focusing on NCDs. Some of the major bottlenecks in the smooth delivery of NCD services in PHC include low disease awareness and possible treatment, poor density of health workers trained to deliver NCDs services, low government spending, inadequate supply of essential drugs for NCDs at primary care level, lack of adequate social health protection scheme for NCD patients; and poor accessibility, and equity and responsiveness of PHC services for NCDs [35]. At national level, however, the acknowledgement of the problem has been evident; Nepal adopted the Multi-Sectoral Action Plan for NCDs (2014–2020) [22], which contains specific national targets and indicators to reduce preventable morbidity, avoidable disability, and premature mortality as a result of major NCDs. The plan highlighted the need for a comprehensive management strategy for NCDs, including risk reduction for prevention, early diagnosis, appropriate management and specific intervention strategies for different levels of health services. However, allocation of resources or activities to address NCDs at the PHC level is minimal, whereas the focus remains on identifying activities for NCD care and prevention at secondary and tertiary care facilities. Early detection through screening and management of NCDs should be the core functions of PHC services, but is lacking. Consequently, often the diagnosis is made after people have severe and hard-to-treat NCD complications. At this late stage, tertiary prevention become as necessity increasing the economic burden on households and worsening health outcomes. As Nepal works toward achieving UHC and SDGs by 2030, there must be a focus around the key principles of PHC service delivery to strengthen quality of care around NCDs. The need to sustain, develop and strengthen PHC is further recognized by the Alma Ata Declaration [20].

To strengthen quality of PHC for NCD management requires allowing more time for health professionals to focus on early detection, diagnosis and treatment of NCDs at primary health centers. Integrating screening for NCDs into routine care should be given a high priority. It is also necessary to ensure availability of simplified guidelines for NCD management in PHC facilities based on the adaptation of WHO’s PEN in LMICs [16]. Health education programmes carried out in PHC need to be assessed and evaluated with a special emphasis on activities for people suffering from chronic diseases. Similarly, community education programmes promoting health awareness, and mitigating the modifiable risk factors need to be strengthened. For instance, efficient community education gives various low-cost but high-impact interventions for the prevention and treatment of NCDs, including advice on tobacco cessation, depleting alcohol consumption, improving diet, and promoting physical activity, which are being referred to as ‘best buys’ [36]. Furthermore, PHC is well positioned to use health technology to enable care in the right place at the right time, but, unfortunately, it remains out-of-reach for many Nepalese due to high cost, lack of training and its perceived value, as described in more details under point 6. The use of mobile health applications, for instance, provides opportunities to engage patients in advancing their health through improved communication and enhanced self-management of chronic conditions such as diabetes, hypertension and cancer.

(5) **Establish strategic information management system**

The prevailing health system in Nepal is still poorly organized with weak health information systems, and inefficient healthcare delivery. For instance, NCD surveillance is ad hoc and fragmented. Data generated are in manual formats at PHC facilities and sent to the secondary level and National Health Management Information System (HMIS) in the form of monthly reports. This manual system of data management often involves duplication of effort. Although the information system for the infectious disease has been established in Nepal, the health information system for NCDs needs to be strengthened further. It is because reliable data on NCD risk factors and mortality are scarce and not integrated into the HMIS in Nepal. The WHO supported Stepwise Approach to Surveillance (STEPS) surveys [37], have only provided initial data on burden of risk factors of NCDs in the country. There is an urgent need to establish electronic medical records to improve the health system’s capacity to track and trace patients across the health system, resulting in a robust NCD surveillance mechanism, such as data collection, storage, analysis, reporting and dissemination of information. Furthermore, national capacity in epidemiological surveillance and research should be strengthened urgently. There should also be development of local and validated tools and protocols to conduct surveys across the country and assess trends of NCDs as well as their risk factors followed by periodic evaluation.

The development of health information system was catalyzed in the Alma Ata Declaration to enable countries to measure progress in delivering PHC [20]. This was also highlighted at the Political Declaration of the recent UN High Level Meeting on NCDs [38] urging member states to integrate NCD surveillance with existing surveys and surveillance systems and monitoring systems, as well as into existing national health information systems. Strengthening surveillance and establishing robust integrated information management system can reduce the gaps in evidence for addressing the burden of NCDs and reduce preventable deaths.

(6) **Strengthening healthcare financing**

Nepal has achieved remarkable improvement in health indicators over recent decades, despite limited fiscal space and poor resource allocation to health section. It achieved Millennium Development Goal 4 and 5 on reducing maternal and child mortality, even with inadequate health financing. These achievements are matched with a revitalized commitment to UHC, including the passage of the 2017 National Health Insurance Act and 2018 Public Health Act, both of which will contribute to progress toward SDGs [15]. However, the high burden of NCDs, increasing elderly care needs and the growing out of pocket expenditure for chronic diseases has put PHC under pressure and there is an urgent need to identify new financial mechanism to focus on achieving UHC. Healthcare is still underfunded in Nepal while it is losing nearly 6% of its Gross Domestic Product (GDP) annually due to premature deaths and preventable illness [39]. The health sector is also neglected in terms of its share in national budget, with only some 4.5% of the total budget in financial year 2019–20 [40]. Of the government expenditure on health, only 6.4% is for NCDs. Further, payments for health services, including medicines and diagnostic costs are unaffordable for many, especially poor people, and this may result in financial hardship as well as deprive people from obtaining the health services that they need. The latest National Health Accounts revealed that people are paying more out-of-pocket for the management of NCDs and the majority of the spending on healthcare is for pharmaceuticals and medical goods [41]. The heavy economic burden borne by patients should be reduced with the improvement of UHC coverage [42]. The current UHC coverage index of Nepal is 46 out of 100, lower than the median national value for service coverage index (65 out of 100) [43] and the health insurance programme is at its nascent stage [44]. Nepal’s health insurance programme has been planned to be expanded gradually. However, we urge for process and outcome evaluation to learn from its implementation challenges (e.g. low enrollment, low retention, pro-rich bias, unfair pricing, and lack of private sector participation with inadequate private sector participation, and rapid adaption within Nepal’s new federal system.

The Alma Ata Declaration, which was reaffirmed in Astana in 2018, emphasized on the role of PHC in service provision to achieve an ambitious goal of health for all hence UHC [21]. Strong PHC systems are effective to create more equitable access to healthcare services among rural and urban areas and consistent health financing mechanism. There is a need for an innovative social health protection schemes to increase healthcare coverage for all segments of population, and avoid catastrophic health expenditures on the affected families. This will require strengthening risk pooling mechanism, designing appropriate benefit package under health insurance scheme and increasing government resources to health sector. In addition, there should also be focus on increasing political commitment for financing health sector, improving system efficiency, preventing and treating NCDs and reducing out-of-pocket expenses.

## The way forward

NCDs present a serious threat to public health and national development in Nepal. The situation is likely to worsen and the management of NCDs requires health systems to adapt to changing needs and respond to rising demand for health services while also addressing the social, political and commercial determinants of health. The PHC approach to NCD, which is holistic and population-focused, provides a valuable guiding framework to reduce disease morbidity and mortality in a sustainable and effective manner and ensures equity and social justice for all (Table 2). Taking this approach can contribute to achieve SDG target 3.4, which targets reduction in premature NCD mortality by a third by 2030.

## Paper context

Nepal is currently facing a double burden of non-communicable diseases (NCDs) and communicable diseases, with rising trends of NCDs. We propose that the primary healthcare (PHC) approach to NCDs offers a guiding framework to reduce disease morbidity and mortality in a sustainable and effective manner and ensures equity and social justice for all. Taking this approach can contribute to achieve Sustainable Development Goal target 3.4, which targets reduction in premature NCD mortality by a third by 2030.
